# Artificial Crowns

**Published:** 1873-08

**Authors:** B. O. Doyle


### ARTIFICIAL CROWNS.

BY B. O. DOYLE.

*Read before the Kentucky and Indiana Dental Associations, June 6th, 1873.*

It is a fact well known, that of late years most, if not all of the improvements in our profession, have been made in the operative department, and mechanical dentistry has been almost entirely overlooked. Therefore in presenting what few thoughts I may, I will attempt to treat of a subject, belonging partly to mechanical dentistry, or at least requiring some mechanical skill. While old, this subject is one of interest and importance, and at times of great vexation, viz: the insertion of artificial crowns, on the natural root. It is well known, that very frequently we are called upon to exercise our best skill for the preservation of the roots of the incisor and cuspid teeth, after caries has destroyed, or so impaired the crown, as to preclude the possibility of restoring the tooth by filling. In such cases, the insertion of an artificial tooth on a pivot is indicated and no doubt would be more frequently practiced, but for the difficulties and uncertainties, with which we are beset in the performance of this operation as ordinarily done. From the early days of our profession, up to the present time, the practice of inserting pivot teeth on wooden pivots, has been followed; and while many cases have stood the test of time, a large majority have proven utter failures, and the causes for these failures are so well known to all of you, that they scarcely need mention. Wood, no matter how tough and well seasoned, will expand by the absorption of moistures, and in many cases will split the root, or, if this does not occur, it will become so soft and yielding, as to allow the artificial crown to be forced from its proper place in the arch, and thus defeat the end we desire to attain. Another great objection to the use of wooden pivots, is that it is almost impossible to deviate them from a straight line, without greatly impairing their strength. I am fully aware of the many "*pet plans and devices*" which have been recommended, such as filling the interstices with "*Hill's stopping*" "*Guilloi's Cement*" etc., but no matter what aids are used with wooden pivots, the great aim, "*durability*" if not defeated, is at least rendered uncertain.

We have all heard of and some of us have seen another method of inserting artificial teeth, in which plate teeth are back lined with gold plate, and have a pivot of gold soldered thereto, and the cavity in the root, if funneled out by decay, is filled with gold before the tooth is applied. But the objection urged against this method is generally by the patient, and is the great cost, and besides so much time is occupied in the performance of this method as to make it exceedingly irksome to both patient and operator.

What we need in the insertion of pivot teeth is some method, by which we can reasonably hope for success without the expenditure of so much time, and which can be practiced at a reasonable fee, so as to bring the operation within the reach of a majority of our patients, who are anxious to preserve the natural organs to the utmost, but are not willing to pay what might seem to them extravagant fees.

I have practiced the following method for some time, and as it has given every assurance of success, and I have heard of no failures, I will proceed to state it; however, without claiming anything especially original or novel, we will take it for granted, that the root has been properly treated, and the apex and part of the nerve cavity thoroughly filled, and the adjacent parts free from inflammation. The root having been prepared by filling, etc., an ordinary pivot tooth is then selected, and properly fitted. A pivot should then be made of heavy gold plate by forming a tube, being careful, however. not to bring the edges entirely together. This should fit the pivot hole tightly, but without compressing the sides of the tube sufficient to bring the edges together. The metallic pivot is then placed in the tooth and applied to the root being of the proper length &c., the pivot is then secured in the pivot tooth with gum shellac melted over a spirit lamp. The tooth and pivot are then secured in a notch cut in a small piece of soft wood by a thin paste of "*Guilloi's Cement.*" By passing the stick armed with the tooth through the flame of the lamp several times, the setting of the cement will be greatly hastened. A napkin is then placed over the lips, and the cavity in the roots having been thoroughly dried with the hot air syringe, is then partially filled with powdered shellac. The wooden shaft is then taken up, and the tooth and pivot warmed over the flame until they have become warm enough to melt the gum, when the tooth can be forced home with a few light taps of the mallet on the end of the wooden shaft. A few drops of water from the syringe will chill the shellac, and the tooth will be firmly anchored.

The reason why the edges of the metallic tube should not be brought together, is that the expansion of the metal when heated would burst the tooth. The advantage of this method is its simplicity and permanency. It is a well known fact that shellac is insoluble in water, or even dilute alcohol, consequetly the fluids of the mouth have no perceptible effect upon it.

